# Proposal to protect patients and healthcare professionals undergoing elective surgery during COVID-19 outbreak

**DOI:** 10.1186/s43055-021-00478-1

**Published:** 2021-04-06

**Authors:** Gülten Arslan, Kemal Tolga Saraçoğlu, Ömer Aydiner, Recep Demirhan

**Affiliations:** 1 Department of Anesthesiology and Reanimation, University of Health Sciences, Kartal Dr Lütfi Kırdar Training and Research Hospital, Istanbul, Turkey; 2 Department of Radiology, University of Health Sciences, Kartal Dr Lütfi Kırdar Training and Research Hospital, Istanbul, Turkey; 3 Department of Thoracic Surgery, University of Health Sciences, Kartal Dr Lütfi Kırdar Training and Research Hospital, Istanbul, Turkey

## Abstract

**Background:**

The aim of this study was to investigate the reliability of COVID-19 Reporting and Data System (CO-RADS) scale on chest computerized tomography (CT) in addition to the reverse transcriptase-polymerase chain reaction (RT-PCR) test in diagnosis of COVID-19 on patients who will undergo elective surgery to protect patients and healthcare professionals during the intense pandemic period and the correlation between CO-RADS scale and Total Severity Score (TSS).  During the intensive pandemic until normalization, 253 patients aged ≥ 18 years who underwent elective surgery with two negative RT-PCR results within the last 5 days and CO-RADS scale ≤ 3 on chest CT were included in the study. Demographic characteristics, American Society of Anesthesiologists classification, type of anesthesia and surgery, department of clinic, chest CT findings, scale of CO-RADS and TSS on CT, patients’ postoperative hospital stay, follow-up time, and vital status (whether or not COVID-19 disease) at the hospital and discharge information were collected for each patient.

**Results:**

The most frequently calculated CO-RADS score was found to be 1 (73.1%). It was followed by scale of CO-RADS 2 (20.9%). Regarding TSS, the most common TSS for the right and left lungs was 0 (91.7% and 92.5%, respectively). COVID-19 was not detected in any of the patients who were hospitalized for an average of 4.9 ± 6.4 days and followed-up for an average of 14.3 ± 8.2 days. It was observed that the CO-RADS score and TSS were positively and moderately correlated with each other (*p* < 0.001).

**Conclusion:**

It was concluded that in early diagnostic of COVID-19, chest CT scans serve like a complementary diagnostic method in addition to RT-PCR testing to keep safe both the patients and health professionals and the scale of CO-RADS and TSS on CT are valuable in correlation with each other.

## Background

SARS CoV2 (severe acute respiratory syndrome-coronavirus 2) is an unusual virus which had been seen as the cause of coronavirus disease 2019 (COVID-19). It had started in China at the last month of 2019 and had dispersed all around the world in a brief duration of time [1]. Our country has naturally been affected by this pandemic and the first official case was announced on March 11, 2020.

As same as what the whole world had done, our Ministry of Health issued a circular to minimize the density in health institutions and reduce the burden on healthcare professionals [2]. This indicated that elective surgeries should be planned for a more convenient date for efficient and rational use of health resources in the extraordinary conditions caused by the pandemic and it should also be planned to minimize the possibility of transmission between patients and healthcare professionals during this process.

In this period, planning the necessary surgeries which improve the life functions and quality, oncological-traumatological surgeries, and performing them with least risk gained even more importance.

Our hospital is located in the largest, densely populated, industrialized city in our country. Due to its location, which is located on main roads, the access to the hospital can be done with ease and the hospital provides a large, comprehensive oncology center. While serving as a huge pandemic hospital, we also had to continue mandatory elective surgeries until normalization.

To minimize the risks for healthcare professionals and patients undergoing elective surgery, some decisions were taken by evaluating the possibilities of our institution and working synchronously with the National Scientific Committee. According to the protocol in our hospital, it was sufficient that RT-PCR tests were negative twice by nasopharyngeal swab within 5 days with an interval of 24–48 h in patients undergoing elective surgery such as malignancy and trauma surgery at the beginning of the pandemic. As it had been observed in the literature [3], upon the observation of COVID-19 in a few of patients at the early postoperative period due to high false-negative results of RT-PCR, the Scientific Committee decided to take and evaluate chest CT with RT-PCR test on patients to be operated in order to protect the healthcare professionals and patients.

In our study, we aimed to evaluate the patients undergoing elective surgery who do not have the diagnose of COVID-19 with negative RT-PCR test and COVID-19 Reporting and Data System (CO-RADS) scale [4] on chest CT. The primary end point of the study was the reliability of CO-RADS scale on chest CT in addition to the RT-PCR test in diagnosis of COVID-19 on patients who will undergo elective surgery during the intense pandemic. The secondary endpoint of the study was to show the demographic characteristics, distribution according to clinical and surgical types of patients undergoing elective surgery, and the correlation between CO-RADS scale and TSS [5] on chest CT.

## Methods

This was conducted as a single center, cohort study at our University Hospital.

This study was approved by the Ministry of Health and Ethics Committee of our University (Ethics Committee decision no: 2020/514/178/4, date: 27 May 2020) and was in accordance with the revised declaration of Helsinki. Written informed consent collected from the participants.

Until normalization (from April 14 to June 1, 2020), during the intense COVID-19 pandemic period, 253 patients aged 18 and over who are undergoing elective surgery which got the result negative twice in RT-PCR test by assay of nasopharyngeal swabs within 5 days in 24–48 h intervals and CO-RADS scale ≤ 3 on non-contrast chest CT were included in the study. Emergency and cesarean surgeries, surgeries performed under local anesthesia, and surgeries involving children under 18 years were not included in the study.

### Chest CT scan

All CT scans for SARS-CoV-2 pneumonia screening were performed with two scanners (128 section Philips ingenuity and 16-section Toshiba Alexion) without contrast material. CT was performed with the patient at the end of inspiration. CT scan parameters: X-ray tube parameters—120 kVp; tube current modulation—120–380 mAs; detector configuration—64 × 0.625 mm or 16 × 0.625 mm; rotation time—0.5–0.7 s; section thickness—5 mm; and pitch—0.984. Reconstruction kernel was lung with thickness and interval of 0.625 mm. All images were viewed in both lung (width, 1200 HU; level, 700 HU) and mediastinal (width, 350 HU; level, 40 HU) settings. The radiographers of 20 years of experience who was blinded to other clinical information were given the task of reviewing the chest CT scans independently and randomly.

### Data collection and definitions

The CT findings were commented using the lung window setting. The CT scans were evaluated, for the presence and distribution of the following abnormalities: (a) ground-glass opacities (GGO); (b) nodules (centrilobular, perilymphatic, or random in distribution); (c) linear density interlobular septal thickening, intralobular septal line, parenchymal bands; (d) crazy paving; (e) consolidations (unilateral, bilateral, multilobular); (f) architectural distortion, or traction bronchiectasis; (g) pleural effusion; (h) lymphadenopathy; (i) air bronchogram; and (j) white lung (defined as diffuse consolidations in a large area of the lung that look like the lung is turning white on CT imaging).

The overall anatomic distribution (subsegmental, segmental, lobar), zonal predominance (upper, middle, lower lung; central, middle, or peripheral location), and extent (focal, multifocal, and diffuse) of the lesions were also recorded.

The standardized reporting system for suspected patients of COVID-19 infection is named as CO-RADS which was developed by Dutch Radiological Society for moderate to high prevalence setting. CO-RADS scoring is based on CT images. The severity or level of suspicion of the infection is listed from very low or CO-RADS 1 up to very high or CO-RADS 5. Two additional classifications, respectively, describe a technically inadequate exam (CO-RADS 0) and SARS-CoV-2 infection as proven by positive RT-PCR at the time of examination (CO-RADS 6) (Table 1) [4].

**Table 1:** Scales of COVID-19 Reporting and Data System CO-RADS (CO-RADS) and the corresponding level of suspicion for pulmonary involvement in COVID-19 (4)

Scales	Level of suspicion for pulmonary involvement of COVID-19	
CO-RADS 0	Not interpretable	Scan technically insufficient for assigning a score
CO-RADS 1	Very low	Normal or non-infectious
CO-RADS 2	Low	Typical for other infection but not COVID-19
CO-RADS 3	Equivocal/unsure	Features compatible with COVID-19, but also other diseases
CO-RADS 4	High	Suspicious for COVID-19
CO-RADS 5	Very high	Typical for COVID-19
CO-RADS 6	Proven	RT-PCR positive for SARS Cov-2

Total Severity Score is calculated by determining percentages for each of the five involved lobes (5):

1. < 5% involvement
2. 5–25% involvement
3. 26–49% involvement
4. 50–75% involvement
5. > 75% involvement

The Total Severity Score calculated as the sum of the individual lobar scores and is scored from 0 (no involvement) to 25 (maximum involvement), when all the five lobes are more than 75% involved [5].

Variables collected included demographics (age, gender), American Society of Anesthesiologists (ASA) classification, type of anesthesia (sedation, general, spinal, epidural, combined spinal-epidural, axillary, etc.), type of surgery (major, moderate, minor) and clinic (general surgery, orthopedics and traumatology, etc.), chest CT findings (presence of GGO, nodules, interlobular septal thickening, intralobular septal line, parenchymal bands, consolidations, architectural distortion, traction bronchiectasis, pleural effusion, lymphadenopathy, air bronchogram, white lung, crazy paving) and CO-RADS scale and TSS, patients’ postoperative hospital stay, and follow-up time. Vital status (whether they developed COVID-19 disease) at the hospital and hospital discharge were collected for each patient. The data were extracted from electronic medical records. The clinical outcomes of these patients were followed up for 14 days. If the patients stayed in the hospital for less than 14 days, their clinical status in relation to COVID-19 was being asked by phone.

### Statistical analysis

IBM® SPSS® (Statistical Package for the Social Sciences) Statistics version 23 were used for the analyzing tool, thus demographic characteristics and collected data of patients were entered to this tool. All values are expressed as mean, maximum, and minimum; percentage values were used for qualitative variables. Normal distributions were reported as mean ± SD. For correlation analysis between CT scores, Pearson correlation coefficient (*r*) was used, assuming the data was normally distributed. If the correlation coefficient was positive, there was a positive relationship between the two variables (an increase in one was associated with an increase in the other). It was assumed that if the coefficient value (*r* value) is < 0.2, it is a very weak relationship; if it is between 0.2 and 0.4, it is a weak relationship; between 0.4 and 0.6 is a moderate relationship; and between 0.6 and 0.8 is considered a high relationship and very high if it is greater than 0.8. If the correlation relationship is moderate or higher, a scatter/dot scatter plot was created and the *r*^2^ coefficient was obtained. The level of statistical significance was set at *p* < 0.05.

## Results

We reviewed patients undergoing elective surgeries at our hospital in the course of the intense COVID-19 pandemic period until normalization. The analysis of results was based on 253 patients who met all inclusion criteria. The age range was 19 to 89 years, and average age was 52.7 ± 16.6 years; 59.68% of the patients were male (Table 2). The most common ASA score was ASA III (*n* = 143, 56.5%). The most frequent type of anesthesia was general anesthesia (*n* = 134, 53%), followed by spinal anesthesia (*n* = 63, 24.9%), procedural sedation (*n* = 45, 17.8%), and axillary block (*n* = 11, 4.3%).

**Table 2:** Demographic and clinical data of patients

Variables	Data
Age, (mean years ± SD)	52.7 ± 16.6
Gender, *n* (%)	
Male	151 (59.7%)
Female	102 (40.3%)
ASA physical status, *n* (%)	
I	35 (13.8%)
II	143 (56.5%)
III	74 (29.2%)
IV	1 (0.4%)
Type of anesthesia, *n* (%)	
Procedural Sedation	45 (17.8%)
General anesthesia	134 (53.0%)
Spinal anesthesia	63 (24.9%)
Axillary block	11 (4.3%)
Type of surgery, *n* (%)	
Minor surgery	41 (16.2%)
Middle surgery	27 (10.7%)
Major surgery	185 (73.1%)
Clinics of surgery, *n* (%)	
Thoracic surgery	29 (11.5%)
General surgery	76 (30.0%)
Plastic and reconstructive surgery	29 (11.5%)
Orthopedic and trauma surgery	72 (28.5%)
Gynecology and obstetrics surgery	11 (4.3%)
Neurosurgery	7 (2.8%)
Ear nose throat surgery	16 (6.3%)
Urologic surgery	13 (5.1%)

Data are *n* or mean ± SD

*ASA* American Society of Anesthesiologists

In the present study, the most common clinical presentation for patients undergoing anesthesia was general surgery (*n* = 76, 30.0%), followed by orthopedic and trauma surgery clinics (*n* = 72, 28.5%). While 185 (73.1%) of the patients underwent major surgery, 41 (16.2%) of them had minor surgery, and 27 (10.7%) had moderate surgery. The patients’ demographic and clinical data are shown in Table 2.

Thirteen (5.1%) patients had bilateral lung involvement, 14 (5.5%) showed unilateral distribution, and 9 (4.3%) showed multilober distribution of CT abnormalities (Table 3). When the distribution was examined, 9 (3.6%) patients had peripheral, 4 (1.6%) patients had posterior, and 4 (1.6%) patients had central parenchymal disease. We observed that the most common localization was the lower side (*n* = 16, 6.3%). The most common patterns seen on chest CT were consolidation [16 (6.3%) patients], pleural effusion [18 (7.1%) patients], ground-glass opacity [7 (2.8%) patients], lymphadenopathy [4 (1.6%) patients], and crazy-paving pattern [2 (0.8%) patients]. Septal thickening, masses, nodules, traction bronchiectasis, cavitation, and calcifications were not observed in our cases.

**Table 3:** Distribution of COVID-19 Reporting and Data System CO-RADS (CO-RADS) scale, Total severity score (TSS), and CT findings

Variables	Data
CO-RADS, *n* (%)	
0	1 (0.4%)
1	185 (73.1%)
2	53 (20.9%)
3	14 (5.5%)
Total severity score, *n* (%)	
0	227 (89.7%)
1	5 (2.0%)
2	5 (2.0%)
3	5 (2.0%)
4	6 (2.4%)
5	3 (1.2%)
≥ 6	2 (0.8%)
Parenchymal involvement, *n* (%)	
Bilateral involvement	13 (5.1%)
Unilateral involvement	14 (5.5%)
Multilobar involvement	9 (3.6%)
Distribution region, *n* (%)	
Peripheral distribution	9 (3.6%)
Posterior distribution	4 (1.6%)
Central distribution	4 (1.6%)
Localization of the distribution, *n* (%)	
Upper	5 (2.0%)
Middle	6 (2.4%)
Lower	16 (6.3%)
Diffuse	8 (3.2%)
Parenchymal state, *n* (%)	
Consolidation	16 (6.3%)
Ground-glass opacification	7 (2.8%)
Crazy paving	2 (0.8%)
Other CT findings, *n* (%)	
Pleural effusion	18 (7.1%)
Mediastinal lymphadenopathy	4 (1.6%)

**Fig. 1:**
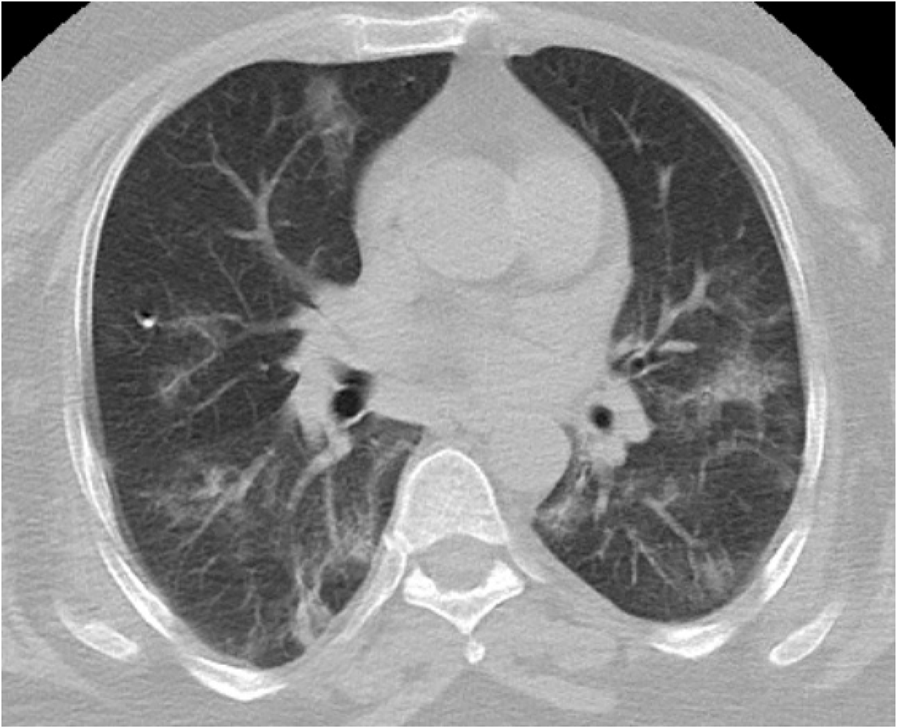
Bilateral patchy consolidation and ground glass opacities more pronounced in the lower lobes and peripheral-CO-RADS 5. Total Severity Index: 6, 40% involvement

**Fig. 2:**
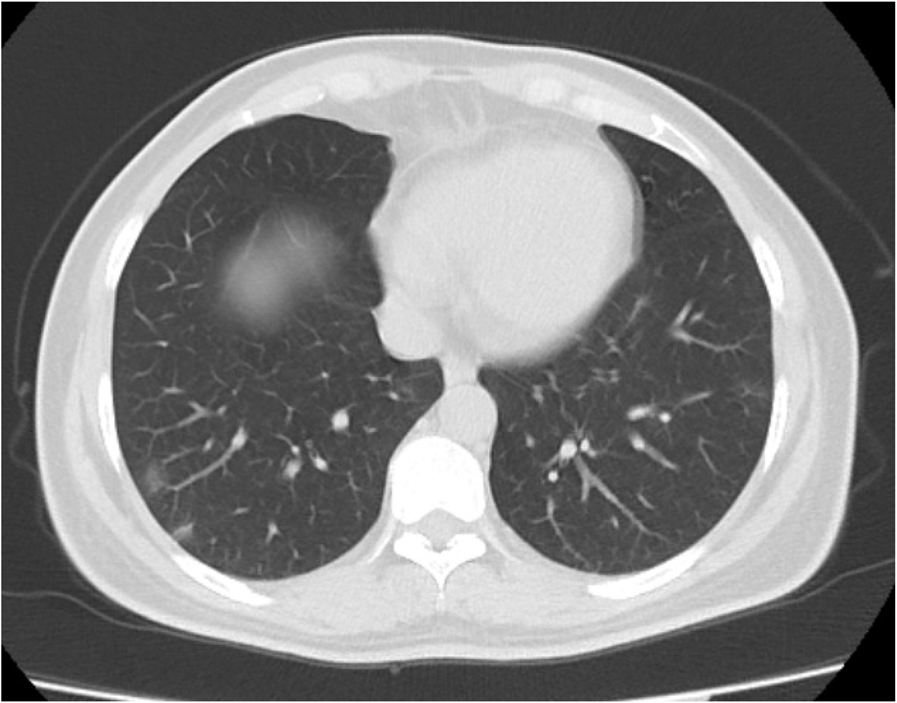
There are ground glass opacities in lower lobes-CO-RADS 4

**Fig. 3:**
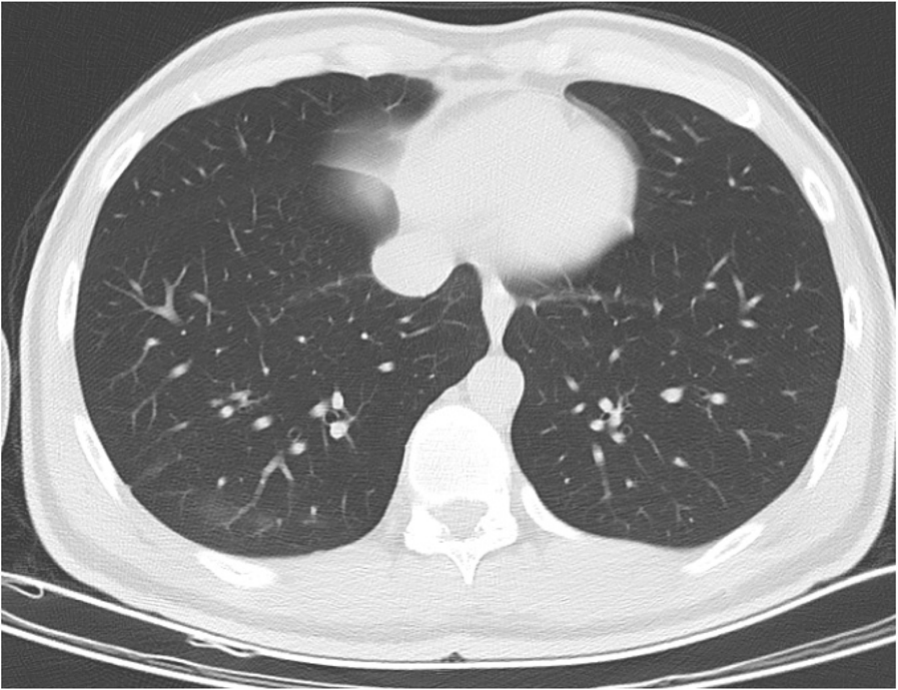
Unifocal GGO in right lower lobe-CO-RADS 3. COVID-19 unsure or indeterminate

**Fig. 4:**
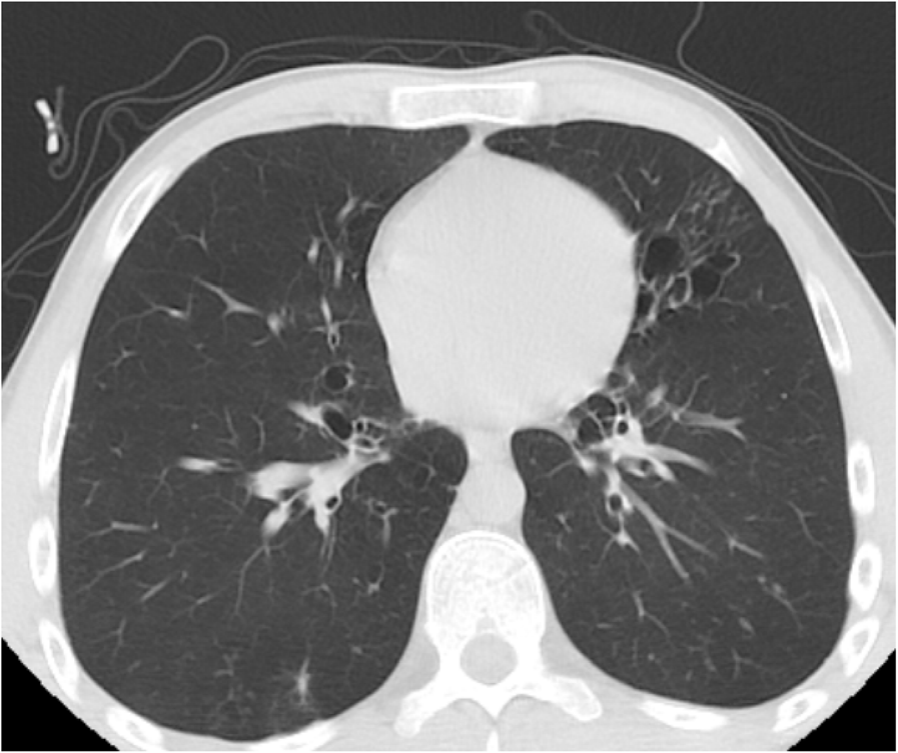
No typical signs of COVID-19The CT-image shows bronchiectasis, bronchial wall thickening—CO-RADS 2. There are no ground glass opacities

**Fig. 5:**
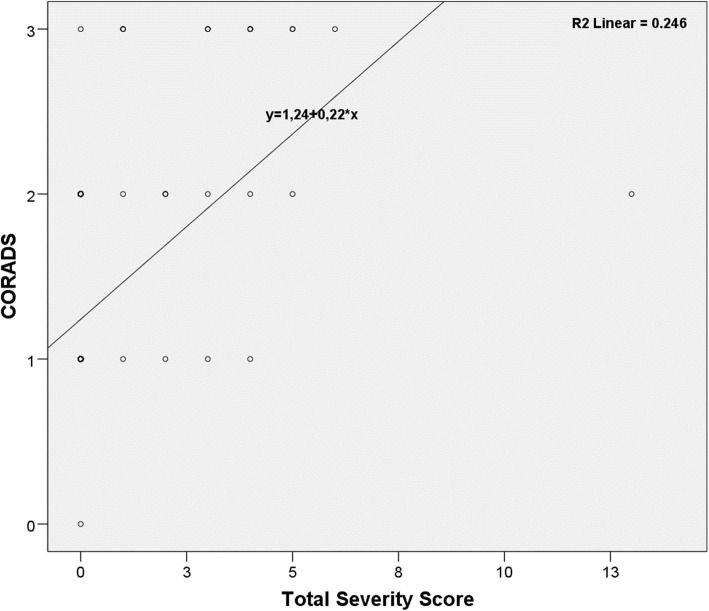
Correlation scatter graph between CO-RADS scale and Total Severity Score

The most frequently calculated CO-RADS score was found to be 1 (*n* = 185, 73.1%). This was followed by CO-RADS scores of 2 (*n* = 53, 20.9%). Regarding TSS, the most common severity score for the right and left lungs was 0 (*n* = 232, 91.7% and *n* = 234, 92.5%, respectively). The data obtained based on the radiological appearance of the patients are shown in Table 3. Figures including various CT images defining the CO-RADS and TSI scores are shown in Figs. 1, 2, 3, and 4. It had been observed that the CO-RADS scale and TSS were positively and moderately correlated with each other and this relationship was statistically significant (Pearson correlation coefficient *r* = 0.496, *p* < 0.001) (Fig. 5).

COVID-19 was not detected in any of the patients who were hospitalized for an average of 4.9 ± 6.4 days (range 1–45 days) and followed-up for an average of 14.3 ± 8.2 days (range 12–64 days).

## Discussion

With an increase in the dispersement of the unusual COVID-19 throughout the world led to fully discontinuance of non-urgent elective surgeries. Although elective surgeries decreased until normalization in our country, we cannot clearly say that it is over. Together with this, the COVID-19 crisis had led to unexpected challenges in the acute management of the crisis. Therefore, there was no standardization about what to do with elective cases in any country. In our literature search, a gold standard clinical algorithm was not found to increase patient safety in elective surgery and to minimize cross-contamination between health workers and patients during the COVID-19 pandemic up to normalization. It was observed that the guidelines published for the management of surgical patients during the pandemic were based only on expert opinion.

It is known that surgery may disrupt the immune system and accelerate disease by triggering an early systematic inflammatory response in patients who undergo surgery during the incubation period of COVID-19 [6]. Lei et al. [7] reported retrospective studies which included 34 patients with a history of direct exposure in the city of Wuhan and no COVID-19 symptoms before surgery and stated that SARS-Cov-2 infection was confirmed by laboratory testing immediately after completion of surgery. The same researchers also showed that 15 (44.1%) patients should be admitted to ICU during disease progression, 7 (20.5%) patients died after admission to ICU, and that duration and size of surgery may be risk factors for poor prognosis. As a result, they noted that surgery can accelerate and exacerbate the progression of COVID-19 disease.

Nucleic acid testing is the gold standard method for confirming SARS-CoV-2 infection and detecting viruses [8]. However, high false-negative results of nucleic acid tests for SARS-CoV-2 were reported for the most used diagnostic tool for COVID-19 screening—the RT-PCR assay using various swab samples [9–11]. It is being reported that these rates increase up to 20–40% (3). In a 56-year-old patient with high fever, Hao et al. [12] reported that three consecutive samples were negative for the SARS-CoV-2, but the patient was diagnosed based on clinical and chest CT findings, and a subsequent 4th RT-PCR test was positive. Specificity of test results are variable because various factors can affect sensitivity. These include source, timing, quality of sample collection, test kit quality, and characteristic features of the patients [3, 13]. Thus, many reports are now advising that the diagnosis of COVID-19 should include CT images together with PCR testing [3, 14]. At the same time, many researchers reported that CT scans are more sensitive to COVID-19 than RT-PCR tests and these were used as standard practice in disease diagnosis until recently [3, 15–17]. Ai et al. [3] reported that chest CT may be considered to be used in the detection of epidemic areas which can be a primary tool for detection of COVID-19.

Because of the false negativity of PCR tests and lower sensitivity of chest radiographs, 100% specificity of chest [18], chest CT with low-dose was also used as a complementary diagnostic approach based on the joint decision of the scientific committee and anesthesia, infection diseases, and surgery clinics at our hospital in the early phase of the COVID-19 outbreak. For reasons we had mentioned above, we decided to evaluate the CO-RADS scale in chest CT in addition to the RT-PCR test in patients who will undergo elective surgery to protect patients and healthcare workers during the Covid-19 intense pandemic period. In addition, TSS, another radiological scoring, and its correlation with the CORADS were also evaluated.CO-RADS was developed in the early period of COVID-19 by the Dutch Radiological Society as a classification system to assess the suspected lung involvement inCOVID-19 on chest CT, and to provide easy and standardized communication [19].

Prokop et al. [19] reported that the distinctive power of CO-RADS for diagnosing COVID-19 during the pandemic was high, with a mean area under the ROC (receiver operating characteristics) curve of 0.91 (95% CI 0.85–0.97) for predicting RT-PCR and 0.95 (95% CI 0.91–0.99) for clinical diagnosis. Also, the false-negative rate for CO-RADS 1 was 5.6% and the false-positive rate for CO-RADS 5 was 0.3%. In accordance with the results of our study, they revealed that it is very suitable in clinical use for the early period of the COVID-19 pandemic. Salahi et al. [20] also observed that CO-RADS facilitated the diagnosis and management of COVID-19 patients in their studies based on imaging data from 37 studies.

TSS is a scoring system used to evaluate the severity of pulmonary involvement in COVID-19 patients. Yang et al. [21] analyzed the thorax CT scans of 102 patients with COVID-19 confirmed by positive RT-PCR; they found that TSS was higher in severe cases and a TSS threshold of 19.5 could identify severe COVID-19, with a sensitivity of 83.3% and a specificity of 94%. As a result, they reported that chest TSS could be used to rapidly identify COVID-19 patients.

In our study, we also observed a positive and moderate correlation between CO-RADS scale and TSS on chest CT. However, we did not observe any related studies in our literature review. Our opinion is that the usage of both scoring scales may increase the safety level if used to support each other.

In addition, we observed that 6 of the patients who were not operated due to CO-RADS 4 and 5 on chest CT had RT-PCR test positivity within the first 5 days, and several cases were admitted to ICU.

### Limitations

We need studies with more samples of patients since our study included limited amount of patients. We postponed the operations of cases with CO-RADS scores of 4 and 5 on chest CT; however, we were only able to obtain information about the prognoses of patients who were admitted to the intensive care unit and followed up by us. We do not have information about patients being followed on their wards or sent home. Perhaps, the number of patients who developed COVID-19 in the early period among the patients who were not taken into operation was more.

## Conclusion

A negative RT-PCR result does not always provide evidence that the virus is not present in that patient; therefore, it was concluded that the addition of chest CT scans as a complementary diagnostic method to the RT-PCR test, which has a low accuracy rate in the early diagnosis of COVID-19 at the onset of the pandemic, may be beneficial to protect patients and healthcare professionals. In addition, we found that on chest CT, CO-RADS and TSS scores are valuable in correlating with each other in confirming the diagnosis of COVID-19, and these scores can be used safely until methods that provide higher accuracy, better predictive power, and more usability emerge.

## Data Availability

All data are publicly available or listed in the results of the paper.

## Abbreviations

SARS CoV2: Severe acute respiratory syndrome-coronavirus 2
CO-RADS: COVID-19 Reporting and Data System
CT: Computerized tomography
TSS: Total severity score
RT-PCR: Reverse transcriptase-polymerase chain reaction
ASA: American Society of Anesthesiologists
COVID-19: Coronavirus disease 2019
GGO: Ground-Glass Opacities
SPSS: Statistical Package for the Social Sciences
ROC: Receiver operating characteristics
